# On the characteristics of reporting ADL limitations and formal LTC usage across Europe

**DOI:** 10.1007/s13385-020-00242-1

**Published:** 2020-07-16

**Authors:** Michel Fuino, Iegor Rudnytskyi, Joël Wagner

**Affiliations:** 1 Department of Actuarial Science, University of Lausanne, Quartier Chamberonne - Extranef, 1015 Lausanne, Switzerland; 2 Swiss Finance Institute, University of Lausanne, Lausanne, Switzerland

**Keywords:** Long-term care, Sociodemographic study, Medical factors, Care policies

## Abstract

The increase in the proportion of elderly people in most industrialized countries triggers higher demand for long-term care (LTC) associated with limitations in activities of daily living (ADL). The aim of this research is to derive the drivers affecting the probability of reporting limitations in ADL and the probability of demanding formal LTC, e.g., personal care and services in domestic tasks. By using the most recent wave of a cross-national European survey on individuals aged over 50 years (SHARE, wave 6), we develop econometric models for identifying the effect of demographic, social and medical factors on ADL limitations and formal LTC along five conjectures. On the one hand, we analyze functional limitations and we find that characteristics such as the age, the gender, the wealth status and the education level influence the probability to report limitations. Further, while we find that pathologies significantly increase the probability to become dependent in general, the effect of cancer is lower. On the other hand, we find again an influence of the demographic and social factors on the probability to use formal LTC. We emphasize on the decrease in the probability due to the presence of the partner in the household, in particular for housekeeping tasks. This is less the case for help related with personal care. In addition, we note that pathologies such as cancer have no influence on the probability to report formal LTC while others like mental and Parkinson diseases highly increase it. We find that elderly living in countries with LTC family care schemes report less formal care than in others. This indicates the importance of LTC policies. Finally, we validate the robustness of our results by applying the models to data from earlier waves of the survey. Our findings give insights for the underwriting standards to be used in future LTC insurance products and for the design of LTC policy environments across Europe.

## Introduction

Handling the forthcoming high number of elderly and in particular the financing threat and infrastructure needs due to the demand for long-term care (LTC) is at the foreground of many policy debates in Europe [38]. In this context, more and more developed countries consider LTC as a new social risk [32]. For example, since the eighties, France considers including the dependence of elderly as a fifth risk of the social security. LTC characterizes the help provided to elderly in need of assistance with the activities of daily living (ADL), namely, bathing, dressing, using the toilet, transferring in and out of a bed or a chair, continence and feeding [65]. Such care is mainly delivered to individuals aged over 65 years [3] with prevalence rates rising exponentially after the age of 80 years [47]. Typically, two types of care, at-home and institutional care, are distinguished. At-home LTC represents the care an elderly receives in his own house while institutional care refers to the one delivered in a specialized institution. While the first type relates to care received upon request, the latter corresponds to 24-h supervision in a specialized infrastructure including accommodation and comes at higher costs. Three major questions arise from LTC [37]. First, the threat of a financial burden stems from the importance of these costs. As things stand, no State can finance the upcoming burden without modifying current social insurance schemes. Further, the contribution by households is considered as too high in most developed countries [103] and large parts of the population cannot afford it. The second major question stems from understanding how to appropriately let private long-term care insurance (LTCI) take part in the financing [25, 114]. Most of the reasons yielding the underdevelopment of LTCI come from the individuals’ underestimation of LTC risk [96], from the discouragement by public policies distributing allowances to the ones without coverage [26] and from the help provided by relatives [23]. Finally, the demand of LTC is driven by many factors and subject to measurement issues. While LTC help provided by professionals, namely formal care, is statistically measurable, help provided by relatives, i.e. informal care, mostly stays hidden beneath the surface and is not directly observable.

In this paper, we first study the reporting of limitations in ADL among elderly in Europe for understanding how demographic, social and medical factors affect them. Limitations in ADL are essential in measuring LTC needs and many factors can affect them. Usually, at high ages, women have more chances than men to present functional limitations [47]. The socioeconomic status also plays an important role since higher wealth often comes along with better health [91]. Further differences in dependence stem from the pathology where, e.g., elderly with diabetes, heart failure and high blood pressure have more chance to require help with ADL than others [95]. In a second part, we investigate on the usage of formal LTC, i.e. help provided by professional caregivers. While functional limitations are an objective measure of elderly dependence, the demand for professional LTC and paid-for services strongly depends on the household composition, financial means and personal believes [102]. In a household, the partner is commonly the first provider of informal care [89]. In that sense, the number of children can also reduce the demand for formal LTC. As part of their social responsibility, children are often caregivers. However, other important factors such as the distance of the parent’s house and the closeness to their parent can play an important role [31, 100]. Our empirical approach builds on data coming from thirteen European countries available in the sixth wave of the Survey of Health, Ageing and Retirement in Europe (SHARE [16]). This European cross-national survey on the health of the population aged over 50 years is conducted every two years, and the data contains information on European countries over the period from 2004 onwards.

Our main results quantify the relevance of demographic, social and medical factors on both the probability to report limitations in ADL and formal LTC usage in Europe. From the cross-national study, we find that demographic factors such as the age and the gender have a strong influence on ADL limitations. For ages above 80 years, women are more likely to present limitations than men are. Further, poorer health conditions such as presenting a body mass index away from normal weight as well as being diagnosed with mental, Parkinson, cancer, musculoskeletal system and other physical diseases increase the probability to be dependent. We observe that mental and Parkinson diseases come along with more limitations when compared to other pathologies. Our study also highlights the role of wealth and education in defining elderly health. With higher education, higher wealth and living together with their partner, elderly tend to report fewer ADL limitations. When detailing our results by type of ADL, we observe that the presence of the partner in the household mostly reduces the claiming of difficulties with bathing. Moreover, when considering the probability to report formal LTC, we find that elderly living with their partner require significantly fewer professional services highlighting the importance of informal care. This effect is particularly affecting domestic tasks while for personal care the presence of the partner does not reduce the claiming behavior. Another interesting outcome is related to the country’s specific LTC policy. Countries with family care schemes, like Italy or Spain, rely more extensively on family members for delivering LTC and appear in our study to strongly decrease the probability for requesting formal LTC. Finally, by considering pathologies, we note that formal LTC is also more often required in cases where mental and Parkinson diseases are diagnosed. We observe that, while some diseases increase the number of limitations in ADL, they do not necessarily entail more formal LTC.

The remainder of this article is organized as follows: Sect. 2 describes the LTC schemes found in Europe and presents five research conjectures that guide the development of the paper. In Sect. 3, we detail the available data and lay out descriptive statistics on the demographic, social and medical factors that we use. Further, we report on the number of available observations by types of ADL and of formal LTC. In Sect. 4, we introduce the econometric models, present the results of their application on the data and discuss the outcomes and their robustness. Finally, we conclude in Sect. 5.

## LTC policies across Europe and research hypotheses

### Landscape of the LTC systems in Europe

LTC refers to the care offered to an elderly in need of assistance in ADL. Depending on the country, the assessment of LTC needs relies on different approaches and recent reforms affect the recognized care levels [61]. Nonetheless, they are mostly extensions of the Katz scale (see [65]). In the following, we discuss the LTC schemes found in thirteen European countries that we will later cover with empirical data.

France, Germany and Belgium share common characteristics in their LTC policy. In France, the LTC expenses are financed by both the government and private insurance. The State distributes a benefit named “Allocation Personnalisée d’Autonomie” to elderly over 60 years in need of LTC regardless of their wealth [29, 30]. The evaluation of the needs is based on a scale combining both instrumental (IADL, including using the phone, using transportation, taking medication and managing money, see for example [18]) and physical ADL (“Autonomie Gérontologie Groupes Iso-Ressources”, see [1]). In Germany, LTC is part of the fifth pillar of social security and included in the statutory health insurance system. Since benefits do not cover the full LTC costs, individuals can buy supplementary private insurance providing additional benefits. Similarly to the assessment found in France, elderly are eligible for receiving benefits in case of difficulties with ADL and IADL. For a long time three care levels were distinguished; in 2017, the system has changed and now uses a five-care levels scale [79]. Belgium is characterized by a highly developed formal LTC scheme complemented by informal care from the family. The system is universal in the sense that the federal compulsory health insurance provides benefits to the whole population. The benefits are means-tested and calculated upon a combination of ADL and IADL scales. In addition, a separate scheme that pays supplementary cash benefits is available in the Flemish region. In the Flemish care insurance, the LTC assessment includes cognitive and social measurements in addition to ADL and IADL scales [109].

Considering the Mediterranean countries, we note similarities in the systems of Spain, Italy and Greece. Common characteristics include the scarce development of private health insurance solutions and the important place of the help from family members [7, 27, 62]. The LTC system in Spain is composed by a universal allowance scheme covering the whole population and classifying eligible elderly along three dependency levels, mild, moderate and severe, related to the number of limitations in ADL. Only moderately and severely dependent individuals can claim benefits. They can choose between cash or in-kind benefits. While selecting in-kind benefits leads to higher allowances, cash is often preferred since it provides a way to remunerate the significant share of informal care delivered by the family. In addition, local authorities provide further benefits subject to means test. In Italy, the LTC scheme is organized at the State, regional and municipalities levels. The acuity of the dependence is defined along severity scales that differ with the region of residence [53, 104]. The “indemnità di accompagnamento” paid only to severely dependent elderly is the most important cash benefit managed by the Italian social security. Equivalently to Spain, there are other benefits paid by municipalities with eligibility conditions changing by location. The Greek LTC public system provides in-kind and cash benefits to elderly over 65 years showing significant difficulties in performing ADL and IADL as well as cognitive impairment [56]. Due to the limitations in formal care services (Open and Day Care Centers, KAPI/KIFI), the government fosters informal help from families through tax reductions [33].

Sweden and Denmark share analogous LTC schemes with both of them offering universal tax-financed coverage to the whole population. The major characteristic of Scandinavian LTC schemes is to ensure that everyone has equal access to care services irrespective of wealth status or place of residence [69]. In Sweden, LTC is regulated at the national level with municipalities being responsible for financing and providing care. Because of the highly developed formal care system, informal care is only perceived as a secondary mean. Measurement of acuity is made through a set of different scales based on ADL and IADL [50, 84]. Benefits are paid in-kind by reimbursing LTC expenses and extra cash benefits are offered to family members providing informal care. The Danish LTC system is similar with the exception that the distinction between institutional and home care is less clear than in Sweden [64].

Central Eastern European countries such as the Czech Republic, Estonia and Slovenia are characterized by a rather low level of LTC services, little regulation by unified laws and high reliance on families. In Czechia, the LTC system is organized through residential, home and community services as well as hospitals for acute care [86]. Based on the ADL scale, a cash allowance is paid by the State along four levels ranging from light to very heavy dependence. In specific cases, in-kind benefits are also provided by the health care system [99]. The systems found in Estonia and Slovenia are relatively similar. Benefits are mostly paid in cash and management is left to municipalities [87].

Finally, we separately discuss the case of Austria and Switzerland. The LTC system in Austria is surely one of the most developed within the European Union. It covers the whole population, but supplementary benefits are subject to means test. Social insurance pays for a cash care allowance “Pflegegeld” where the amount depends on the number of hours of care required varying along seven levels. Further, a 24-h care support benefit is available for those receiving at-home care under means-test conditions. The system is tax-based and managed at the federal and regional levels [32]. Despite the relatively-well developed formal care system, family still plays a central role for shortening the costs. In Switzerland, the LTC system is poorly developed [47]. Mandatory health insurance supports formal care at-home and provides in kind benefits for specific furniture. An old-age care allowance is paid to all $65+$ elderly in need of LTC irrespective of their wealth. Especially in institutional care, more than 40% of the costs are left to the households while social security helps those who cannot afford it [103].

In Table 1, we summarize for each country the LTC system in place with the type of funding, the organizational levels and the types of functional limitations in ADL and IADL considered for defining dependence levels and benefits. The second column informs about our categorization of the different countries LTC schemes into four groups (family care, State responsibility, subsidiary and none). We describe these classes in the next section (see Conjecture 3).

**Table 1:** LTC schemes across selected European countries

Country	LTC scheme	Funding source	Organizational level	ADL	IADL
Austria	State responsibility	Taxes	Federal and regional	✓	✓
Belgium	Subsidiary	Health insurance	Federal and regional	✓	✓
Czechia	Family care	Health insurance	National, regional and municipal	✓	
Denmark	State responsibility	Taxes	Municipal	✓	✓
Estonia	Family care	Taxes	Regional and municipal	✓	✓
France	Subsidiary	Taxes and private insurance	National	✓	✓
Germany	Subsidiary	Health insurance	Regional and municipal	✓	✓
Greece	Family care	Health insurance	National	✓	✓
Italy	Family care	Taxes	Regional and municipal	✓	
Slovenia	Family care	Taxes	Regional and municipal	✓	
Spain	Family care	Taxes	Regional	✓	
Sweden	State responsibility	Taxes	National and municipal	✓	✓
Switzerland	None	Taxes and health insurance	Federal and regional	✓	

### Research hypotheses

In the following, we derive five conjectures hypothesizing on the characteristics of LTC needs in a sample of European countries. Due to the existing heterogeneity in regulation and in the availability of LTC services, the European continent is a perfect candidate for such study. Taking a cross-country view can provide stronger support to conclusions. The need of LTC is assessed by the measurement of reporting limitations in ADL along the Katz scale. Further, we consider the reporting on the usage of formal LTC services. In our empirical study, we are able to assess these measures throughout selected countries (see Sects. 3, 4).

**Sociodemographic factors** The age and the gender are often used in public health research to predict the need of LTC. Germain et al. [52], find that age and gender are essential drivers for the risk stratification and for explaining health limitations. Similarly, many authors develop models using age and gender to account for the related heterogeneity in insurance pricing (see, e.g., [34, 40, 46, 90]). Nonetheless, due to limitations of the available data, such studies rarely consider medical factors that are often a major reason for the dependence. For example, the occurrence of diseases like Alzheimer and cancer strongly depends on the individuals’ age and gender (see, e.g., [72, 77, 78, 97]). Further, for ages close to life expectancy, the age and the gender are less relevant in shaping LTC needs [115].

In the United States, spouses are often the first provider of at-home care [89]. In case of a spouse’s absence or inability to provide care, adult children and their spouses represent the second layer for informal care. While receiving help form the partner is usual, help from children is less common and is related to social responsibility of children towards their parents [31, 116]. Societal trends linked to globalization induce increased distances between the childrens’ and their parents’ locations conducting to higher use of formal care solutions. [43] show that married American elderly have significantly lower prevalence rates in functional limitations than the non-married. On that basis, we consider that elderly living in a two persons household present lower acuity levels than those living in a single person household. Even if it is only a second layer, the presence of children can strongly reduce the reporting of limitations in ADL. For example, [20] finds that childless elderly are 20.3% to have at least one ADL limitation in comparison to 14.5% for the ones having children not living in the same household and 3.0% for the ones living together with their children. Therefore, we also expect to observe a significant reduction in functional limitations when an elderly has children.

The educational level and the wealth status are often considered as a proxy of the social class of an individual. Freedman et al. [44] bring evidence that higher functional limitations come along with lower education levels and might also drive the demand for formal LTC. On a sample of about six thousand American elderlies observed over the period from 1984 to 1993, the authors find a significant reduction in the prevalence of limitations when educational attainments are higher. This conclusion is also supported by [45] who analyze the effect of many factors including the education level on the frailty of elderly. Their study also shows that wealthier individuals are less likely to present ADL limitations. Many studies link lower financial resources to higher LTC needs [91]. Thereby, a partial explanation for such interaction between wealth and health stems from the level of insurance since wealthier elderly often hold supplementary coverage enhancing access to expensive health care services [76]. However, these findings are contrasted by other studies indicating no causality of wealth on health [75].

Conjecture 1: *Both (a) the probability to report ADL limitations and (b) the probability to report formal LTC usage are significantly affected by demographic (age, gender, body mass index, daily smoking) and social factors (partner in the household, children, wealth status, education level).*

**Pathologies** For a long time, mental and physical diseases have been said to strongly impact healthy limitations [54, 48, 55] and many studies evidence the effect of dementia. Based on a longitudinal study of 407 elderly without dementia or disability, Lau et al. [70] find that limitations in functional capacities are highly related to the occurrence of mental diseases. The case of Alzheimer among elderly is well studied since it is the most common cause of dementia. Barberger-Gateau et al. [5] and Tuokko et al. [106], discuss the correlation between functional limitations and Alzheimer. Diseases affecting bones and strength always lead to limitations. This assertion probably also holds for physical diseases such as cancer, diabetes, heart failure and high blood pressure. For example, Avis and Deimling [2] suggest that cancer affects physical functioning. The same is true for elderly diagnosed with diabetes, heart failure and high blood pressure (see, e.g., [68, 71, 95]). In the discussion of Conjecture 2, we will argue that cancer might lead to fewer functional limitations than other diseases since, while having higher mortality, it does not always imply dependency (see, e.g., [54], where no significant effect of cancer on functional limitations is found).

Conjecture 2: *Medical factors (mental, Parkinson, cancer, musculoskeletal system and other physical diseases) have a strong effect on (a) the probability to report ADL limitations and (b) the probability to report formal LTC usage.*

**LTC schemes** Our third conjecture directly follows from the discussion in Sect. 2.1. Based on the description of the countries, three clusters of LTC systems emerge. “State responsibility” models are LTC systems in which the government plays a central role. Independently of the wealth status, every citizen in need of care is entitled to receive benefits for financing home- and institution-based care. Mostly funded by taxes, the management and the financing of care are left to local authorities. Municipalities must be aware of the care needs and propose suitable solutions. Such models take inspiration from the Scandinavian health system that offers universal public coverage financed to a large part by taxes [64]. Further, formal community care services (home- and institution-based care) are well developed and cover most of the required LTC. Finally, such models present higher LTC expenditures but satisfy well the population’s needs. They also enhance the requirement of care provided by relatives often considered as a secondary solution [4]. The Swedish and the Danish LTC system are part of this category. Further, we also consider the Austrian scheme here although it is a mixture between State responsibility and family care. In fact, “family care” schemes position the family of the dependent elderly as a major actor. Elderly must first request help from their family before relying on State solutions. Further, family members stand as one of the only affordable caregivers inhibiting the development of community services. The government pays limited benefits that are subject to means test and to a minimum acuity level threshold. Such benefits are mostly paid in-cash for facilitating financial retribution to relatives providing care. Family care models are mostly present in Southwestern Europe [24] and to a lesser extent in Central and Eastern Europe (CEE) where most of the population believe that care provided by children is the best option [19]. Spain, Italy, Greece as well as CEE countries mostly hold family care systems [7]. Finally, “subsidiary” models represent a trade-off between State responsibility and family care models. Such schemes are characterized by well-developed community services and by a strong involvement of the family. Elderly in need of care typically have to announce themselves to the State authority which is responsible to manage the individual health path. However, benefits provided by the system are insufficient to overcome the total LTC costs. To curtail the financial pressure, the family is required to participate in the financing and to provide care ([28]). Such models mix both formal and informal care. The subsidiary LTC model is found in Germany, France and Belgium [64]. Finally, Switzerland has to be considered separately since no proper scheme LTC system is developed (classified as “none”). Our classification is close to the one proposed by [83]. Table 1 summarizes the LTC schemes as laid out above.

Conjecture 3: *Both (a) the probability to report ADL limitations and (b) the probability to report formal LTC usage significantly differ by types of LTC schemes. *

**Types of ADL limitations** The scale by [65] determines functional limitations among elderly based on six ADL, namely dressing, bathing, getting in and out of bed, toileting, walking, and eating [6]. Based on medical assessments, LTC dependence follows common patterns. Activities requiring lower extremity strength are affected before upper extremity strength activities [66, 67]. While a clear distinction is often made among functional limitations, e.g., advanced items such as bathing and dressing and basic items such as toileting and feeding [92], only little research is made on the effect of sociodemographic factors on individual ADL limitations [39]. Iwarsson et al. [60] report results from a survey on adults aged over 75 years carried out in Germany, Hungary, Latvia, Sweden and the UK. Their results reveal that less than 5% report difficulties with feeding while more than 20% report limitations with transferring and bathing. Results from the Assets and Health Dynamics of the Oldest Old Survey (AHEAD) also indicate a hierarchical distinction between ADL. For example, elderly report fewer difficulties with activities such as toileting and eating than with walking and dressing [57].

Conjecture 4: *The probabilities to report limitations along types of ADL are differently affected by demographic, social and medical factors.*

**Formal LTC usage** Formal LTC corresponds to professional help provided to an elderly in need of assistance with ADL. These services are distinguished between personal care delivered by nurses and domestic tasks provided by non-medical staff [21, 105]. For example, in the U.S., according to [42], about 93% elderly require help with transportation while only about 45% need assistance with personal care. Distinguishing between the types of care is important because the lack of caregivers is mostly related to personal care and the required medical staff rather than to domestic tasks [49, 59]. Further, elderly can often rely on their partner for domestic tasks, while they require professional help for personal care [63].

Conjecture 5: *The probabilities to report formal LTC usage along personal care and domestic tasks are differently affected by demographic, social and medical factors.*

## Dataset and descriptive statistics

To explore Conjectures 1 to 5, we rely on econometric models applied on the records of the Survey of Health, Ageing and Retirement in Europe (SHARE). In Sect. 3.1, we describe the main characteristics of the SHARE dataset and discuss the variables of interest. Then, we present the descriptive statistics and lay out the main figures in Sect. 3.2.

### Description of the SHARE dataset

The SHARE dataset provides information on a representative sample of individuals aged 50 years and over living at home (excluding nursing homes and hospitals). Covering twenty-seven European countries, this study is re-conducted every two years and has registered six waves over the period from 2004 to 2018. The records are summarized in modules. Eight modules report original respondents’ answers on behavioral risks, children, consumption attitude, demographics, employment, pensions, peak flow, health care and medical assessment. The other three modules contain generated variables for correspondence to international classification standards on physical and mental health, on education and on social network information. The data collection process is managed by local universities that mandate professional polling firms. The process is broken down into two parts: computer-aided personal interviews (CAPI) and paper and pencil medical questions. In the first part, the elderly is assisted by a purpose-trained employee for reporting answers and measuring health indicators such as grip strength and blood pressure. In the second part, the interviewee has to fill in a form treating more personal questions including pathologies.^1^ The survey results contain detailed information about respondents’ sociodemographic, economic and health characteristics. The individual’s *age*, *gender*, *body mass index* (BMI), smoking habits (*daily smoker*) and *country of residence* are typical demographic factors. The survey also contains information on the number of limitations in ADL an elderly has. In our study, we only consider ages from 65 to 99 years, a range where most dependent elderly are found [3]. With respect to the smoking habits and BMI, the records reveal if the respondent has ever been a daily smoker whereas we cluster BMI responses within the six classes defined by the international scale from the [111], i.e. underweight, normal weight, overweight, moderately obese, severely obese and very severely obese. The country of residence corresponds to one of the twenty-seven European countries within the SHARE dataset. We do not consider behavioral factors such as drinking habits and the loneliness measure since the direction of the causal effect is not clear. For example, medication provided to persons with ADL may prevent alcohol consumption or loneliness may be due to disability.

Regarding the socio-related variables, we first construct a variable *partner in household* for identifying the type of household, i.e. single- and two-persons households. We observe that this factor is highly correlated with the marital status that we do not consider in our sample. Next, we consider the variable of having *children* and we include it as a binary (yes, no) response. Given the substantial difficulties in adding information on the distance to the closest child and the presence of women among children, we exclude them from our study. We introduce the *wealth status* by considering the financial capacity to make ends meet, categorized among four levels, i.e. easily, fairly easily, with some difficulty and with great difficulty. We account for the individuals’ *education level* by using the international standard classification of education (ISCED-97) defined by the UNESCO. The SHARE data records the seven levels of the ISCED-97 scale as well as two other classes (persons still studying and others). In our approach, we eliminate individuals with entries in the class “still studying” and “others” representing less than 2% of the data. Further, we cluster the ISCED-97 entries into three groups. The primary education consists in pre-primary and basic education. The secondary education corresponds to the lower, upper and post-secondary education, while the tertiary education contains the first and second stage of tertiary education [107].

Finally, we consider the physical and mental health status by constructing five medical factors based on the respondents’ answers to the medical questions “Doctor told you had: ...”. This approach is close to the one used in [3] for reporting the effect of diseases on home care utilization among elderly. The *mental diseases* variable is a binary variable that takes the value of one when at least one “yes” appears in the responses to Alzheimer, dementia and emotional disorders. We also consider separately the diagnosis of *Parkinson disease* and *cancer*. Positive responses to hip and femoral fractures, rheumatoid arthritis, osteoarthritis and other fractures are gathered through the *musculoskeletal system diseases* variable. The *other physicals diseases* variable, namely, heart attack, stroke, diabetes, chronic lung disease, cataracts and chronic kidney represent the last cluster.

### Descriptive statistics

Our study focuses on the latest wave (wave 6) published in 2018 that contains answers from $39\,808$ respondents aged between 65 and 99 years. Starting from the raw data, we merge the responses to construct single respondent identifiers. We disregard data from Croatia, Israel, Luxembourg, Poland and Portugal since less than thousand records are available in each of these countries. Due to missing entries in several variables, the inclusion of these countries would lead to the exclusion of many factors of interest. In the end, we remain with 26 331 complete records from the 13 countries discussed in Section 2 (also see Table 2 below). As part of our data processing, we are able to fill some missing entries in wave 6 by recovering invariant information provided by respondents in previous waves. Among the respondents, 22 400 are autonomous elderly while 3 931 report at least one limitation in ADL corresponding to a prevalence rate of 14.9%. Across the countries, this percentage of dependent elderly ranges between 9% and 19%.

**Demographic, social and medical factors** Table 2 reports descriptive statistics on the overall, the autonomous (“Aut.”) and the dependent (“Dep.”) respondents’ data (first three columns in each section of the table). When considering the demographic factors, we note that the panel is mostly composed by elderly younger than 85 years (89.4%) with a higher proportion of dependent individuals among the $80+$. We observe lower shares of men than women and find the largest disparity (63.3% vs 36.7%) among the dependent elderly. The BMI class with the highest number of records is the “overweight” class. However, we find more “severely obese” and “very severely obese” in the population reporting ADL limitations. About 40% have ever smoked daily. While our records are well distributed among the 13 countries, the family care LTC scheme prevails and represents more than half of the records.

**Table 2:** Descriptive statistics on reported ADL limitations and formal LTC usage by demographic, social and medical factors

		Overall	Aut.	Dep.	w/o FC	FC			Overall	Aut.	Dep.	w/o FC	FC
*Demographic factors*													
Age							Country of residence						
65–69	%	29.8	32.3	15.8	20.0	8.6	Austria	%	6.8	6.9	6.3	4.5	9.3
70–74	%	24.2	25.5	16.7	19.8	11.6	Belgium	%	8.9	8.4	11.5	8.2	17.1
75–79	%	20.8	20.7	21.0	22.0	19.3	Czechia	%	9.2	9.1	9.9	12.4	5.8
80–84	%	14.6	13.3	21.6	20.5	23.5	Denmark	%	5.5	5.8	3.5	2.7	5.0
85–89	%	7.6	6.2	16.0	12.5	22.0	Estonia	%	10.1	9.6	12.8	16.4	6.7
90–94	%	2.6	1.8	7.3	4.1	12.7	France	%	7.0	6.7	9.0	7.0	12.3
95–99	%	0.4	0.2	1.6	1.1	2.4	Germany	%	6.9	7.0	6.7	5.4	8.8
**Gender**							Greece	%	7.2	7.4	5.7	6.1	5.0
Male	%	43.0	44.1	36.7	40.9	29.7	Italy	%	8.9	8.9	8.5	9.5	6.8
Female	%	57.0	55.9	63.3	59.1	70.3	Slovenia	%	6.7	6.6	7.5	10.2	3.2
**Body mass index**							Spain	%	9.3	9.2	9.8	9.5	10.3
Underweight	%	1.3	1.0	2.7	1.9	3.9	Sweden	%	8.2	8.7	5.5	5.4	5.6
Normal weight	%	34.6	35.5	29.6	26.6	34.6	Switzerland	%	5.3	5.7	3.3	2.7	4.1
Overweight	%	42.2	43.2	36.3	37.5	34.2	**LTC scheme**						
Moderately obese	%	16.6	15.9	20.3	21.8	17.9	State responsibility	%	20.5	21.5	15.3	12.6	19.9
Severely obese	%	4.2	3.6	8.0	8.9	6.5	Family care	%	51.4	50.8	54.2	64.0	37.8
Very severely obese	%	1.1	0.8	3.1	3.3	2.9	Subsidiary	%	22.8	22.0	27.2	20.6	38.2
**Daily smoker**							None	%	5.3	5.7	3.3	2.7	4.1
Yes	%	41.3	42.1	37.0	38.1	35.2							
*Social factors*													
**Partner in household**							**Education level**						
Yes	%	60.1	62.4	46.6	55.1	32.4	Primary	%	28.2	26.6	37.7	36.0	40.7
**Children**							Secondary	%	51.9	52.3	49.7	51.3	46.9
Yes	%	90.3	90.4	89.5	92.3	85.0	Tertiary	%	19.9	21.1	12.6	12.7	12.4
**Wealth status**													
High	%	35.5	37.2	26.0	23.2	30.7							
Mid-high	%	29.1	29.4	26.9	25.8	28.8							
Mid-low	%	25.4	24.5	30.9	33.4	26.7							
Low	%	10.0	8.9	16.2	17.6	13.8							

		Overall	Aut.	Dep.	w/o FC	FC			Overall	Aut.	Dep.	w/o FC	FC
*Medical factors*													
**Mental diseases**							**Musculoskeletal system diseases**						
Yes	%	8.3	6.0	21.1	18.4	25.6	Yes	%	33.6	30.0	54.4	52.2	58.5
**Parkinson disease**							**Other physical diseases**						
Yes	%	1.2	0.6	4.5	3.7	5.9	Yes	%	42.1	38.3	63.8	62.5	65.9
**Cancer**													
Yes	%	5.2	4.8	7.5	7.1	8.0							
**Nb. of individuals**	*N*	26 331	22 400	3931	2461	1470		*N*	26 331	22 400	3931	2461	1470

“Aut.” stands for autonomous, “Dep.” for dependent, “w/o FC” for without formal care and “FC” for with formal care

Regarding the social factors, 60% report living with their partner, 90% have children and about 65% are found in the mid-high and high wealth status classes. More than 70% have at least a secondary education background. When considering the dependent population, the main differences arise from the lower share reporting to have their partner in the household (46.6%), from the higher shares in the low (16.2%) and mid-low (30.9%) wealth status classes and from the lower share with tertiary education (12.6%). Finally, when considering medical factors (multiple diseases can be reported), few autonomous individuals report having mental, Parkinson or cancer diseases. Overall, the shares of elderly reporting musculoskeletal system (30.0%) and other physical (38.3%) diseases is significantly higher. We observe higher shares for all diseases when considering the dependent population: more than half announce musculoskeletal system (54.4%) and other physical (63.8%) diseases; 21.1% present mental diseases, 4.5% and 7.5% report Parkinson and cancer diseases, respectively. These findings give first insights on the difference between the autonomous and the dependent populations. In fact, individuals that report limitations in ADL are, in comparison to the autonomous population, older, with a higher BMI and more to live alone. Expectedly, they report more diseases highlighting the importance of considering pathologies when discussing ADL limitations.

Table 2 also reports statistics on formal LTC used by elderly presenting functional limitations (last two columns). Among the 3931 dependent elderly, 2461 have not reported using any professional services (“w/o FC”) while 1470 have used formal care (“FC”). The distribution of the observations along age classes is noticeably different when comparing dependent elderly using professional services with the others. The share of elderly using formal care increases at ages $80+$. For example, they are 22.0% in the segment from 85 to 89 years, to require professional services, which compares to 12.5% of the respondents reporting not using professional help. Further, records on elderly requiring formal help are composed by 70.3% of female and 29.7% of male. This high share of female stems from the higher female life expectancy of dependent elderly (see, e.g., [73]). While BMI and smoking habits do not show important differences between both groups, we note strong differences among LTC schemes. Dependent respondents announcing the usage of qualified help are 19.9% living in a State responsibility scheme, 37.8% living in a family care scheme and 38.2% living in a subsidiary scheme. Conversely, the share of elderly not using formal care is much higher in countries with family care policies. The shares are 64.0%, 12.6%, 20.6% and 2.7% for dependent persons not reporting professional help, respectively. Considering social and medical factors, we observe most significant differences with the presence of the partner in the household and diagnosed mental diseases. A share of 55.1% of dependent elderly not using formal care live with their partner while only 32.4% are in two persons households among the professional care takers. Dependent elderly reporting formal care usage also have a higher share of mental diseases (25.6%).

**Table 3:** Descriptive statistics on the reported limitations in specific ADL

ADL	*N*	Share
Dressing	2686	68.3%
Bathing	2145	54.6%
Getting in and out of bed	1238	31.5%
Toileting	833	21.2%
Walking	746	19.0%
Eating	650	16.5%

Shares are based on 3931 elderly reporting limitations in ADL

**Types of ADL limitations** Among the 3 931 elderly reporting limitations in ADL, we present their distribution along the six ADL in Table 3. A single individual can report more than one limitation. We find that the ADL that has been reported the most is dressing (68.3% of the dependent elderly), followed by bathing (54.6%), getting in and out of bed (31.5%), toileting (21.2%), walking (19.0%) and eating (16.5%). This ranking is similar to the one reported by [42]. Although they solely study people diagnosed with dementia coming with more limitations, they find that more than half of their sample is in need of assistance with bathing and dressing while eating, toileting and walking show significantly lower shares.

**Formal LTC usage** In Table 4, we detail on the reported formal LTC usage by the 3931 dependent elderly. Overall, 1470 (37.4%) report using professional help: 29.0% receive help for domestic tasks and 20.4% get personal care. A lower share uses meals-on-wheels services (9.8%), professional help with other activities (9.3%) and seasonally stays in nursing homes (2.3%). The other 2461 dependent elderly (62.6%), although reporting limitations in ADL, do not report using any professional services. Recall that the SHARE data only records answers from elderly living at home. This explains the limited usage of nursing home services.

**Table 4:** Descriptive statistics on the reported formal care usage of specific services

Services	*N*	Share
*With formal care*	1470	37.4%
Domestic tasks	1139	29.0%
Personal care	803	20.4%
Meals-on-wheels	387	9.8%
Other activities	366	9.3%
Nursing home	92	2.3%
*Without formal care*	2461	62.6%

Shares are based on $3\,931$ elderly reporting limitations with ADL

## Econometric models and results

In Sect. 4.1, we develop econometric models to study the reporting of limitations in ADL and of formal care usage. Further, we present our results and link them to the research hypotheses in Sect. 4.2. In Sect. 4.3, we discuss the validation of the conjectures and study the robustness.

### Model framework

**Explanatory variables** We are interested in understanding the effect of various factors on the probability to report limitations in ADL and on the probability to report formal LTC. For this purpose, we consider the demographic, social and medical factors introduced in Sect. 3.2. The individual’s age (*AG*) is a numeric variable running from 65 to 99 years. Further, we account for nine binary variables as follows. Four sociodemographic variables indicate whether the respondent is a man or a woman (*GE*), has ever smoked daily (*SM*), is living with a partner in the same household (*HH*) and has children (*CH*). Five medical factors indicate whether the respondent has mental (*MD*), Parkinson (*PA*), cancer (*CR*), musculoskeletal system (*MS*) or other physical diseases (*PD*). In these variables, we chose the “no” answer respectively “male” for *GE* as the baseline category. Choosing “no” for the baseline allows for a direct interpretation of the “yes” category, i.e. having a certain disease, smoking habit, partner in household or children. Finally, we consider four sociodemographic categorical variables giving information on the respondent’s body mass index (*BM*), wealth status (*WS*), education level (*ED*) and country of residence’s LTC scheme (*SC*). The range of values taken by the categorical variables are discussed in Sect. 3.1 and summarized in Table 5. For each of the variables we define the baseline category and consider “normal weight”, “low” wealth status, “primary” education level and “none” for the country’s LTC scheme. With that choice the baseline either refers to the lowest category value respectively the healthy situation in the case of the body mass index. We include all the above variables in our econometric models and use the notation ${\textit{\textbf{X}}}$ to refer to the set of variables reported in Table 5.

Since our objective is to develop models explaining the effect of the above variables and not to make a prediction, we keep all variables in each model and study their statistical significance. However, to avoid too complex models, we only consider relevant interactions that improve our model along the Bayesian information criterion (BIC) values using a backward step-wise selection algorithm and present the detailed results of this approach in Table 13 in the Appendix. Throughout the various models tested (see below and Table 6), the retained interaction terms along BIC are the same. In the models explaining the probability to report ADL limitations, only the gender and age interaction appears relevant. In the probability to report formal LTC models, the interactions of the partner in the household with both age and gender as well as of the children with gender interaction are retained.

**Table 5:** Description and values of the independent variables included in ${\textit{\textbf{X}}}$

Variables	Description	Values
*AG*	Age	from 65 to 99
*GE*	Gender	male, female
*BM*	Body mass index	6 classes from underweight to very severely obese
*SM*	Daily smoker	yes, no
*HH*	Partner in household	yes, no
*CD*	Children	yes, no
*WL*	Wealth status	high, mid-high, mid-low, low
*ED*	Education level	primary, secondary, tertiary
*MD*	Mental diseases	yes, no
*PA*	Parkinson disease	yes, no
*CR*	Cancer	yes, no
*MS*	Musculoskeletal system diseases	yes, no
*PD*	Other physical diseases	yes, no
*SC*	LTC scheme	State responsibility, family care, subsidiary, none

**Model selection** To investigate the probability to report limitations in ADL, we define six binary variables $\ell ^j$ corresponding to the ADL. These variables take the value of one when a limitation in the $j^{\text {th}}$ ADL, namely, dressing ($j=1$), walking ($j=2$), bathing ($j=3$), eating ($j=4$), getting in and out of bed ($j=5$) and toileting ($j=6$) is reported. It takes the value of zero otherwise. For studying the reporting of formal care usage, we construct two binary variables $f^k$ identifying the reporting of help for personal care ($k=1$) and with domestic tasks ($k=2$). We do not analyze the other formal care services (meals-on-wheels ($k=3$), other activities ($k=4$) and nursing ($k=5$)) given their lower prevalence (less than 400 observations, see Table 4). With these notations, we can formally write out the dependent variables of interest. The probability to report at least one limitation in any ADL can be written as ${\mathbb {P}}(\sum \nolimits _j \ell ^j_i>0)$ where *i* refers to the $i^{\text {th}}$ individual. The same probability for a specific ADL *j* is ${\mathbb {P}}(\ell ^j_i=1)$. The probability to report the usage of at least one formal LTC service is ${\mathbb {P}}(\sum \nolimits _k f^k_i>0)$ and the one related to a specific service *k* is ${\mathbb {P}}(f^k_i=1)$.

A common method for estimating probabilities with econometric models is to use logistic and probit regression models. Denominated as binomial generalized linear models (GLM), these methods differ through the link function used to relate the dependent variable to the independent variables. While the logistic model uses the *logit* function, the *probit* model assumes a quantile of the standard normal distribution as link function. For comparison, we also consider a linear regression model. To decide which model yields the best fit, we apply four performance measures. They are the log-likelihood, the deviance, the in-sample area under the curve (AUC) and AUC based on 5-fold cross-validation. Better models present higher log-likelihood and AUC but lower deviance. For selecting the best model, we consider both the probability to report at least one limitation in ADL, ${\mathbb {P}}(\sum \nolimits _j \ell ^j_i>0)$, and the probability to report the usage of at least one formal LTC service, ${\mathbb {P}}(f^k_i=1)$ for an individual *i*. We report the performance measures in both cases in Table 6. We find that the probit model performs better than the logit function for ${\mathbb {P}}(\sum \nolimits _j \ell ^j_i>0)$ and we therefore use the probit model when studying the ADL limitations. Using the same statistical procedure, we determine which model fits best to analyze the probability to report a specific ADL limitation ${\mathbb {P}}(\ell ^j_i=1)$. Here, we also find that the probit model offers the best fit. With regard to ${\mathbb {P}}(\sum \nolimits _k f^k_i>0)$, we find that the logistic model is better suited (see Table 6). When considering the probability to report formal LTC with personal care or with domestic tasks, the logistic regression model also outperforms the other models.^2^ Further, we decide not to include weights usually used for controlling sampling characteristics, validity of causal relationships and statistical inference. In fact, one should be very careful when considering adding weights and such a choice must be double-checked with appropriate diagnostics [98, 110]. For example, in our case, the inclusion of the calibrated cross-sectional weights (as available in SHARE) leads to a decrease in the *in-sample* AUC from 0.7883 (cf. first column of Table 6) to 0.7859. This is the case because SHARE individual cross-sectional weights are computed in each country and wave across gender-groups and regional areas [15]. Since most of this information is already accounted through our explanatory variables, considering the SHARE individual cross-sectional weights becomes purposeless.^3^

**Table 6:** Performance measures for probit and logit link functions on the probability to report limitations in ADL and usage of formal LTC

	${\mathbb {P}}(\sum \nolimits _j \ell ^j_i>0)$		${\mathbb {P}}(\sum \nolimits _k f^k_i>0)$	
	Probit	Logit	Probit	Logit
*In-sample*				
Log-likelihood	−**9156.91**	$-9167.86$	$-2189.99$	−**2185.57**
Deviance	**18313.83**	$18\,335.72$	4379.98	**4371.14**
AUC	**0.7883**	0.7880	0.7600	**0.7603**
*5-fold cross-validation*				
AUC	**0.7872**	0.7867	0.8027	**0.8027**

**Specification of the models** Following on the above discussion, we introduce two models to study the probability to report ADL limitations. In model (1), we study the probability to report at least one limitation with ADL which can be written as

1 $$\begin{aligned} \text {probit}\left [ {\mathbb {P}}\left( \sum \nolimits _j \ell ^j_i>0\right) \right] = \alpha + \varvec{\beta } {\textit{\textbf{X}}}_i + \beta _{AG\cdot GE } AG_i \cdot GE_i, \end{aligned}$$

where $\alpha$ is the intercept, $\varvec{\beta }$ are the vectors of regression coefficients for the variables ${\textit{\textbf{X}}}_i$ (see Table 5) and $\beta _{AG \cdot GE}$ is the regression coefficient for the age-gender interaction term for a respondent *i*. In model (2), we detail the first model by identifying the relation between the covariates and specific functional limitations *j*. The considered regression equations for $j=1,\ldots , 6$ are:

2 $$\begin{aligned} \text {probit}\left [ {\mathbb {P}}\left( \ell ^j_i=1\right) \right] = \alpha + \varvec{\beta } {\textit{\textbf{X}}}_i + \beta _{AG \cdot GE} AG_i \cdot GE_i. \end{aligned}$$

We evaluate this second model separately for each type of ADL, i.e. $j=1,\ldots , 6$.

Similarly, we develop two models for studying the probability to report formal LTC usage. Through model (3), we analyze the probability of using at least one formal LTC service:

3 $$\begin{aligned} \text {logit}\left [ {\mathbb {P}}\left( \sum \nolimits _k f^k_i>0\right) \right] = \alpha + \varvec{\beta } \varvec{X_i} + \beta _{HH \cdot AG} HH_i \cdot AG_i + \beta _{HH \cdot GE} HH_i \cdot GE_i + \beta _{CD \cdot GE} CD_i \cdot GE_i. \end{aligned}$$

Again, $\alpha$ represents the intercept and $\varvec{\beta }$ the regression coefficients for the covariates ${\textit{\textbf{X}}}_i$. The coefficients $\beta _{HH \cdot AG}$, $\beta _{HH \cdot GE}$ and $\beta _{CD \cdot GE}$ are related to the three interaction terms considered. We refine model (3) by considering separate types of formal LTC in model (4), i.e. for $k=1,2$:

4 $$\begin{aligned} \text {logit}\left [ {\mathbb {P}}\left( f^k_i=1\right) \right] = \alpha + \varvec{\beta } \varvec{X_i} + \beta _{HH \cdot AG} HH_i \cdot AG_i + \beta _{HH \cdot GE} HH_i \cdot GE_i + \beta _{CD \cdot GE} CD_i \cdot GE_i. \end{aligned}$$

This model is evaluated twice, separately for the probabilities to report use of personal care $(k=1)$ and help with domestic tasks $(k=2)$.

### Results and discussion

In this section, we report the obtained results when applying models (1) to (4) on the data described in Sect. 3. We discuss our findings along the Conjectures 1 to 5 introduced in Sect. 2.2. The numerical outcomes are presented in Tables 7 and 8. In each table, we report the coefficient estimates with their standard error in parentheses. We assess the statistical significance through *p*-values using the notations “.” for a *p*-value below 0.1, “*” for a *p*-value below 0.05, “**” for a *p*-value below 0.01 and “***” for a *p*-value lower than 0.001. The study of ADL limitations in models (1) and (2) is based on the full set of 26 331 observations. The usage of LTC services in models (3) and (4) is analyzed on the 3 931 records related to dependent elderly (cf. Table 2). In addition to the regression results, we study the deviance decrease for each of the variables included in models (1) and (3). In the Appendix, we report the percentage of deviance decrease in Table 14. Further, confusion matrices for all models can be found in Table 15.

#### Probability to report limitations in ADL

Following the results presented in Table 7, we discuss our findings along the conjectures.

**Table 7:** Results for regression models (1) and (2) on the probability to report limitations in ADL

Model	Model (1)	Model (2)					
	Dependent	Dressing	Walking	Bathing	Eating	In/out of bed	Toileting
**Intercept**	$-3.914$ (.195)***	$-3.541$ (.210)***	$-4.581$ (.348)***	$-5.229$ (.252)***	$-4.324$ (.351)***	$-4.318$ (.297)***	$-4.416$ (.333)***
**Age**	0.033 (.002)***	0.025 (.003)***	0.029 (.004)***	0.044 (.003)***	0.028 (.004)***	0.027 (.003)***	0.028 (.004)***
**Gender (baseline: Male)**							
Female	$-1.402$ (.226)***	$-1.411$ (.246)***	$-1.343$ (.385)***	$-1.299$ (.286)***	$-1.451$ (.405)***	$-0.694$ (.327)*	$-1.171$ (.374)**
**Gender** $\times$ **Age**	0.018 (0.003)***	0.017 (.003)***	0.017 (.005)***	0.018 (.004)***	0.018 (.005)***	0.010 (.004)*	0.016 (.005)**
**Body mass index (baseline: Normal weight)**							
Underweight	0.437 (.082)***	0.469 (.108)***	0.458 (.089)***	0.440 (.110)***	0.402 (.102)***	0.357 (.111)**	0.357 (.111)**
Overweight	0.043 (.025).	0.060 (.028)*	$-0.059$ (.042)	$-0.053$ (.031).	$-0.102$ (.044)*	0.041 (.036)	$-0.016$ (.041)
Moderately obese	0.266 (.031)***	0.323 (.034)***	0.039 (.053)	0.104 (.038)**	$-0.039$ (.056)	0.198 (.044)***	0.098 (.051).
Severely obese	0.573 (.047)***	0.625 (.050)***	0.254 (.077)**	0.302 (.058)***	0.020 (.091)	0.354 (.065)***	0.328 (.074)***
Very severely obese	0.983 (.080)***	1.136 (.081)***	0.735 (.110)***	0.799 (.090)***	0.450 (.130)***	0.757 (.098)***	0.628 (.114)***
**Daily smoker (baseline: No)**							
Yes	0.041 (0.023).	0.034 (.025)	0.028 (.040)	0.081 (.029)**	$-0.001$ (.042)	$-0.016$ (.034)	0.051 (.039)
**Partner in household (baseline: No)**							
Yes	$-0.084$ (.024)***	$-0.021$ (.026)	$-0.042$ (.040)	$-0.138$ (.029)***	$-0.056$ (.042)	$-0.032$ (.033)	$-0.027$ (.039)
**Children (baseline: No)**							
Yes	0.019 (.035)	0.030 (.039)	$-0.112$ (.055)*	$-0.012$ (.042)	0.039 (.062)	$-0.037$ (.049)	$-0.107$ (.054)*
**Wealth status (baseline: Low)**							
Mid-low	$-0.150$ (.035)***	$-0.152$ (.038)***	$-0.179$ (.054)***	$-0.132$ (.041)**	$-0.185$ (.058)**	$-0.145$ (.045)**	$-0.171$ (.052)**
Mid-high	$-0.294$ (.036)***	$-0.296$ (.039)***	$-0.262$ (.056)***	$-0.261$ (.043)***	$-0.268$ (.061)***	$-0.261$ (.048)***	$-0.251$ (.055)***
High	$-0.385$ (.038)***	$-0.379$ (.041)***	$-0.309$ (.060)***	$-0.341$ (.045)***	$-0.333$ (.064)***	$-0.346$ (.051)***	$-0.325$ (.058)***
**Education level (baseline: Primary)**							
Secondary	0.013 (.024)	0.027 (.027)	$-0.033$ (.039)	$-0.019$ (.029)	0.038 (.042)	0.032 (.033)	$-0.002$ (.038)
Tertiary	$-0.139$ (.034)***	$-0.107$ (.037)**	$-0.232$ (.061)***	$-0.234$ (.044)***	$-0.170$ (.064)**	$-0.104$ (.050)*	$-0.140$ (.058)*
**Mental diseases (baseline: No)**							
Yes	0.619 (.032)***	0.604 (.033)***	0.510 (.045)***	0.692 (.034)***	0.675 (.045)***	0.609 (.038)***	0.673 (.042)***
**Parkinson disease (baseline: No)**							
Yes	1.046 (.076)***	0.952 (.076)***	0.639 (.095)***	0.847 (.080)***	0.898 (.089)***	0.939 (.082)***	0.835 (.088)***
**Cancer (baseline: No)**							
Yes	0.219 (.043)***	0.162 (.047)***	0.203 (.067)**	0.275 (.050)***	0.248 (.069)***	0.295 (.056)***	0.257 (.064)***
**Musculoskeletal system diseases (baseline: No)**							
Yes	0.400 (.022)***	0.383 (.024)***	0.196 (.037)***	0.243(.027)***	0.104 (.039)**	0.248 (.031)***	0.205 (.036)***
**Other physical diseases (baseline: No)**							
Yes	0.356 (.021)***	0.326 (.024)***	0.277 (.037)***	0.378 (.027)***	0.316 (.039)***	0.315 (.031)***	0.281 (.036)***
**LTC scheme (baseline: None)**							
State responsibility	0.080 (.057)	0.096 (.063)	0.446 (.136)**	0.091 (.078)	0.191 (.110).	0.222 (.103)*	0.242 (.111)*
Family care	0.058 (.055)	0.011 (.060)	0.499 (.132)***	0.215 (.074)**	0.103 (.107)	0.423 (.098)***	0.287 (.107)**
Subsidiary	0.256 (.055)***	0.193 (.061)**	0.418 (.134)**	0.343 (.075)***	0.149 (.109)	0.256 (.101)*	0.159 (.110)
*N*	$26\,331$	$26\,331$	$26\,331$	$26\,331$	$26\,331$	$26\,331$	$26\,331$

$$p < 0.1$$, *$$p < 0.05$$, **$$p < 0.01$$, ***$$p < 0.001$$

**Conjecture 1a** When considering the results obtained for model (1) on the probability to report at least one limitation in ADL, we find that both age and gender are important. The coefficient for the age effect is positive and statistically significant ($\beta _{AG}=0.033$) indicating that the probability to report limitations in ADL increases with the age. This probability appears, at first glance, to be lower for female than for male ($\beta _{GE}=-1.402$). However, this only holds true for ages below 78 years. In fact, when adding the age and gender interaction, we note that at higher ages, women present a higher probability when compared to men. This finding is in line with the conclusions by [101] who find that women have more limitations in ADL than men at ages above 77 years. Focusing on the BMI, we observe that the probability to be dependent significantly increases when deviating from normal weight. Most extreme results are found for very severely obese persons ($\beta _{BM}=0.983$). Having ever smoked daily does not influence the probability. Further, we find that the presence of the partner in the household significantly decreases the probability to report at least one limitation ($\beta _{HH}=-0.084$). In fact, spouses are often the first providers of care and their presence in the household surely leads to an underestimation of personal frailty [89]. Indeed, partners actively participate in domestic tasks and provide help with other activities. Our result also relates to the finding in [82] stating that elderly living together with their partner are less likely to require help in an institution. Next, we remark that the sole information of having children does not impact the probability of being limited in ADL. Even if a non-significant effect of children is also found by [108], we mitigate such assertion since we believe that more precise data like, e.g., the distance between parents and children needs to be considered [26]. Our outcomes reveal that wealthier elderly are more likely to be autonomous. This is particularly visible in elderly from the high wealth class (baseline) when compared to the low-income class ($\beta _{WL}=-0.385$). Following on that, we also note that individuals with a higher education level report fewer difficulties with ADL [9, 35, 41, 44, 45]. As an answer to Conjecture 1a, we conclude that the age, the gender, the BMI, the presence of the partner in the household, the wealth status and the education level significantly affect the probability to report at least one limitation in ADL.

**Conjecture 2a** The medical information about the diagnosis of mental, Parkinson, cancer, musculoskeletal and other physical diseases are significantly related to the reporting of functional limitations. Among the considered diseases, Parkinson has the strongest impact ($\beta _{PA}=1.046$), reducing the motor system of the human body and yielding four main symptoms: tremor (shaking), rigidity (stiffness), bradykinesia (slowness of movements) and postural instability [80]. These symptoms directly induce disabilities of performing ADL and entail dependence. Mental diseases have the second highest effect on the occurrence of limitations ($\beta _{MD}=0.619$). As discussed in Sect. 2.2, Alzheimer is among the most diagnosed mental diseases and leads to strong functional impairments [5, 106]. According to the [112], mental disorders are a major contributor to the global burden of diseases and in particular among elderly. Further, the presence of musculoskeletal system and other physical diseases increases the probability since they directly relate to functional limitations ($\beta _{MS}=0.400$ and $\beta _{PD}=0.356$). Intuitively, one would expect that physical diseases have a stronger effect on ADL limitations than mental diseases. However, two main components of the factor “other physical diseases” are stroke and heart attack, which rather cause death than long-term disability in aged people. Finally, the effect of cancer is found to be the smallest ($\beta _{CR}=0.219$). Similarly to strokes and heart attacks, cancer more rapidly leads to death when compared to other diseases, sometimes avoiding elderly to experience dependence (see, e.g., [54, 41]).

**Conjecture 3a** In our analysis of the LTC scheme’s effect on the probability to report limitations in ADL, we take Switzerland (“none”) as the baseline for comparison. Given that Switzerland has not developed any particular scheme, it is the perfect reference candidate [103]. From the numerical results, we remark that elderly living in countries with subsidiary schemes, i.e. Belgium, France and Germany, show significantly higher probability to report limitations in ADL. Such result may be mostly due to Belgium and France gathering a higher share of dependent persons (11.5% and 9.0%, see Table 2 in Sect. 3.2). For both State responsibility and family care schemes, we do not observe any significant difference in the probability of reporting functional limitations when compared to Switzerland. This highlights a quite similar pattern and rather shows that the reporting of ADL limitations is not influenced by the LTC scheme. In fact, this could be expected since the population health should not relate to a specific LTC policy.

**Conjecture 4** Our fourth conjecture investigates on potential differences existing along ADL types. For all the types of ADL, the probability to report limitations increases at higher ages, for females and for a lower wealth status as well as for elderlies with mental, Parkinson, cancer, musculoskeletal system and other physical diseases. However, we note differences in the other factors. For example, persons defined as moderately obese have difficulties with dressing, bathing and getting in and out of bed while that characteristic is not significant in walking, eating and toileting. Severely obese persons do not present a significant limitation with eating while very severely obese significantly infer assistance with all activities. Detailing limitations by type of ADL also reveals that the presence of the partner in the household reduces the probability to report limitations with bathing while it has no significant effect on the other activities. Another interesting result comes from the education level. Only persons having a tertiary level of education show a lower probability to report difficulties with walking and bathing activities ($\beta _{ED}=-0.232$ and $\beta _{ED}=-0.234$) when compared to the primary education baseline. Such result may come from the education level being related to the type of profession done during the working life. Indeed, there is strong evidence that blue-collar workers present higher functional limitations at high ages than white-collar workers (see, e.g., [36]). Finally, the LTC schemes affect only heterogeneously the functional limitations impeding a clear interpretation.

#### Probability to report usage of formal LTC

Below we comment on the results displayed in Table 8 along the Conjectures 1b, 2b, 3b and 5 related to the usage of formal care services.

**Table 8:** Results for regression models (3) and (4) on the probability to report usage of formal LTC

Model	Model (3)	Model (4)	
	Formal LTC	Personal care	Domestic tasks
**Intercept**	$-4.719$ (.617)***	$-6.165$ (.714)***	$-5.219$ (.650)***
**Age**	0.062 (.007)***	0.062 (.008)***	0.059 (.007)***
**Gender (baseline: Male)**			
Female	0.766 (.242)**	0.696 (.274)*	0.783 (.255)**
**Body mass index (baseline: Normal weight)**			
Underweight	0.287 (.231)	0.503 (.233)*	0.389 (.234).
Overweight	$-0.099$ (.091)	0.005 (.104)	$-0.135$ (.098)
Moderately obese	$-0.073$ (.109)	$-0.101$ (.128)	$-0.048$ (.118)
Severely obese	$-0.182$ (.153)	$-0.039$ (.184)	0.042 (.162)
Very severely obese	0.249 (.223)	0.498 (.258).	0.317 (.239)
**Daily smoker (baseline: No)**			
Yes	0.127 (.086)	$-0.048$ (.099)	0.157 (.093).
**Partner in household (baseline: No)**			
Yes	$-2.754$ (1.026)**	$-1.369$ (1.011)	$-3.907$ (.957)***
**Partner in household** $\times$ **Age**	0.021 (.011)*	0.011 (.012)	0.030 (.012)*
**Partner in household** $\times$ **Gender**	0.700 (.171)***	0.011 (.200)	1.197 (.189)***
**Children (baseline: No)**			
Yes	0.020 (.208)	0.208 (.247)	0.151 (.230)
**Children** $\times$ **Gender**	$-0.847$ (.256)***	$-0.663$ (.291)*	$-0.821$ (.274)**
**Wealth status (baseline: Low)**			
Mid-low	$-0.150$ (.116)	$-0.044$ (.139)	$-0.228$ (.128).
Mid-high	$-0.014$ (.121)	0.045 (.144)	$-0.032$ (.132)
High	$-0.041$ (.128)	$-0.049$ (.151)	$-0.111$ (.138)
**Education level (baseline: Primary)**			
Secondary	$-0.103$ (.083)	$-0.214$ (.094)*	$-0.047$ (.089)
Tertiary	$-0.067$ (.125)	$-0.301$ (.146)*	0.071 (.134)
**Mental diseases (baseline: No)**			
Yes	0.364 (.090)***	0.502 (.098)***	0.300 (.096)**
**Parkinson disease (baseline: No)**			
Yes	0.517 (.174)**	0.515 (.183)**	0.687 (.181)***
**Cancer (baseline: No)**			
Yes	0.271 (.139).	0.153 (.160)	0.066 (.154)
**Musculoskeletal system diseases (baseline: No)**			
Yes	0.077 (.077)	$-0.141$ (.088)	0.224 (.083)**
**Other physical diseases (baseline: No)**			
Yes	0.212 (.079)**	0.205 (.091)*	0.237 (.085)**
**LTC scheme (baseline: None)**			
State responsibility	$-0.011$ (.216)	0.439 (.243).	0.106 (.227)
Family care	$-1.206$ (.209***	$-0.803$ (.240)***	$-1.210$ (.222)***
Subsidiary	0.190 (.209)	0.236 (.237)	0.356 (.219)
*N*	$3\,931$	$3\,931$	$3\,931$

Note: . $$p < 0.1$$, * $$p < 0.05$$, ** $$p < 0.01$$, *** $$p < 0.001$$

**Conjecture 1b** The results from model (3) show that the probability to report formal LTC usage is positively driven by the individual’s age. Due to the increase in ADL limitations, older individuals have higher chances to request formal LTC ($\beta _{AG}=0.062$). Further, because of their lower mortality, females present higher probability to use formal LTC than males ($\beta _{GE}=0.766$). Focusing on the other sociodemographic factors such as the BMI, the smoking habits, the presence of children, the wealth status and the education level, we observe that they do not significantly affect the probability. Nevertheless, we would have expected a positive effect along higher wealth status or higher education level since these segments might ask for more professional services. In fact, while fewer functional limitations are reported by higher socio-economic classes, in case of dependence, they may hold financial means or supplementary insurance coverage yielding easier access to formal LTC [76]. An interesting result comes from the analysis of the household composition. Persons living with their partner in the household present a significantly lower probability to report formal LTC ($\beta _{HH}=-2.754$). Further, the men’s probability is significantly lower when having their spouse in the household than for women (positive coefficient $\beta _{HH \cdot GE}=0.700$). This confirms that spouses and mostly women are often the first provider of at-home care [89].

**Conjecture 2b** Recall that when analyzing the probability to report functional limitations in the previous section, we have found that diseases yield higher limitations. We are now interested in understanding how diseases can affect the use of formal LTC. Focusing on the values of the different diseases’ coefficients, we find the highest impact and statistical significance for mental diseases ($\beta _{MD}=0.364$) and Parkison ($\beta _{PA}=0.517$). We note a clear distinction between limitations in ADL and usage of formal LTC. While these diseases highly influence the number of functional limitations (cf. Table 7), they do not necessarily entail more formal LTC usage. Indeed, formal care services are often requested in case of diseases that importantly affect functional limitations. Caring for patients with dementia or Parkinson requires complementing informal with formal care especially due to behavioral disturbances [63, 113]. Further, we highlight that, in comparison to the above diseases, less formal care is sought in the case of cancer, musculoskeletal system and other physical diseases ($\beta _{CR}=0.271$, $\beta _{MS}=0.077$ and $\beta _{PD}=0.212$) since those patients can often be better handled by the informal caregivers without involving an unbearable burden in all cases.

**Conjecture 3b** Formal LTC usage can be affected by the type of LTC policy. Our results show that living in family care scheme countries differently affects the probability to use formal LTC than living in places where State responsibility or subsidiary schemes are in place. In fact, we find that elderly living under family LTC schemes have significantly lower probabilities to report formal care than the others ($\beta _{SC}=-1.206$). This outcome becomes even more interesting when we compare it to the results observed in the functional limitations where such scheme has no particular effect on reducing the probability to report limitations (cf. Table 7). Therefore, we can draw various conclusions. First, policies surely affect the delivery of formal care. In family care countries, the State is often explicitly relying on help provided by family members since the development of private services LTC is low (see, e.g., [8, 94] and our discussion in Sect. 2.1). For example, in Italy most of the allowances are paid only to severely dependent elderly forcing families to rely first on informal care if they cannot afford professional care expenses. Second, culture plays an important role [51]. While relying on professional care appears to be common in countries with State responsibility and subsidiary schemes, using formal care services is often avoided in family care systems. Third, the development of specialized infrastructures, their availability, as well as the training of at-home professional caregivers is a condition to the usage of formal LTC. Scarce availability is often an issue in family care systems when formal LTC is less developed.

**Conjecture 5** Our last conjecture is interested in the factors’ differences between the two types of formal LTC, personal care and domestic tasks. In fact, personal care is often both psychologically and mentally more demanding for the informal caregiver than domestic tasks that relate to common household responsibilities. While our results do not show particular differences when comparing the effect of the age, the gender, the BMI, the smoking habits and the wealth status, we observe diverging results in the other factors. Having the partner in the household highly reduces the request of professional services for domestic tasks ($\beta _{HH}=-3.907$). However, this reduction effect is hindered when the elderly requiring LTC is a woman ($\beta _{HH \cdot GE}=1.197$). Similar outcomes are found in the literature on informal LTC. For example, [81] find a higher share of women providing help with ADL than men (e.g., 30% of women with a dependent partner are providing help with bathing, while this ratio decreases to 13% for men). Regarding the education levels, we note that the probability to use personal care is reduced in elderly with a higher education level ($\beta _{ED}=-0.214$ for secondary and $\beta _{ED}=-0.301$ for tertiary education levels, respectively). This effect is not significant for domestic tasks. Looking at the pathologies, we observe that mental diseases and Parkinson increase the probability to request professional help with both personal care and domestic tasks. Being diagnosed with cancer does not influence these probabilities. Further, major differences appear when considering musculoskeletal system diseases and other physical diseases which significantly affect the probability to use help with domestic tasks. Finally, we find that the probability to request formal LTC with personal care and domestic tasks is significantly lower in family care schemes. This effect is stronger for domestic tasks ($\beta _{SC}=-1.210$) than for personal care ($\beta _{SC}=-0.803$). In fact, the offer of services in domestic tasks is broader when compared to personal care since fewer qualifications are needed.

### Validation of conjectures and discussion

Using the results from Sect. 4.2 based on wave 6 data from SHARE, we assess whether the conjectures defined in Sect. 2.2 hold. Then, we extend our validation procedure by comparing results with historical data. In fact, our analysis has been performed so far on the most recent wave 6. To evaluate the robustness of our conclusions, we apply the regression models (1) and (3) as far as possible on previous waves of the SHARE data. This allows us to present results (see also the Appendix) validating or refuting conjectures on the basis of earlier waves. Table 9 presents a summary of our final conclusions on the conjecture with cross-wave comparisons.

**Table 9:** Summary results on the validation of the conjectures through waves 1, 2, 4, 5 and 6

	Wave 6	Wave 5	Wave 4	Wave 2	Wave 1
*Probability to report ADL limitations*					
Conjecture 1a	✓	✓	✓	✓	✓
Conjecture 2a	✓	✓	✓	✓	✓
Conjecture 3a	(✓)	✗	✗	✗	✗
Conjecture 4	✗				
*N*	$26\,331$	$17\,031$	$11\,975$	$7\,274$	$6\,269$
*Probability to report formal LTC usage*					
Conjecture 1b	(✓)	(✓)		(✓)	(✓)
Conjecture 2b	(✓)	✓		✓	✗
Conjecture 3b	✓	✗		✗	✗
Conjecture 5	✓				
*N*	3931	2542	1960	1092	983

“✓” proven, “(✓)” partially proven “✗” refuted. Conjectures 1b, 2b and 3b on the probability to report formal LTC usage cannot be tested in wave 4 since this wave does not report on paid-for services. Conjectures 4 and 5 considering the demographic, social and medical factors cannot be addressed on historical data since the various regressions include too many different definitions among waves making any results hardly comparable

**Assessment with wave 6 data** From the findings in Sects. 4.2.1 and 4.2.2 our conclusions are as follows: the first conjecture questions the effect of sociodemographic factors on (1a) the probability to report ADL limitations and on (1b) the probability to report formal LTC. For the probability to report ADL limitations, we find that, on the exception of smoking habits and the presence of children, all factors have a significant effect, therefore confirming Conjecture 1a. For the probability to report formal LTC, the answer is mitigated because BMI, smoking habits, wealth status and education level appear to have no significant impact. For this reason, we only partially validate the effect of sociodemographic factors in Conjecture 1b. Our second conjecture wonders on the effect of pathologies. We conclude that an individual’s pathology strongly affects functional limitations, thus confirming Conjecture 2a. With less emphasis (not all medical factors have a highly significant effect) our results also yield the validation of Conjecture 2b for pathologies affecting the usage of formal LTC. Regarding Conjectures 3a and 3b, our outcomes yield for partial agreement. For the reporting of functional limitations, subsidiary LTC schemes are the only to produce a difference when compared to countries with no LTC scheme. These results may however be an artefact to the higher prevalence of dependent persons in those countries in our data. We consider partial validation of (3a). For the probability to use formal LTC, elderly living in countries with family care schemes show a significantly and strongly lower probability when compared to the baseline, while living in one of the two other LTC scheme countries has no impact. The fourth conjecture questions the importance of analyzing ADL separately. Our study does not reveal important differences in the factors affecting the probability to report any of the ADL individually. Our main comments concern the difference in the effect of the BMI depending on the activity and on the presence of the partner in the household that essentially reduces the probability for bathing limitations. Finally, Conjecture 5 considers the distinction of formal care between personal care and domestic tasks. We find clear evidence on the role of the partner in the household reducing the probability to require help with domestic tasks while no significant effect appears when considering personal care. The education level is significantly reducing the probability to report professional help usage for personal care while it does not affect the one for domestic tasks. Further, living in a country with a family care policy has a higher reduction effect on the probability to report help for domestic tasks than for personal tasks. From these observations, we conclude on the importance to distinguish among types of formal LTC and validate Conjecture 5. We summarize the above conclusions in the column entitled “Wave 6” in Table 9.

**Assessment with historical data** To further confirm our hypotheses and evaluate the robustness of our results, we use data from previous SHARE studies where waves 1, 2, 4 and 5 (years 2004, 2006, 2012, 2015) provide some data on elderly reporting limitations in ADL and usage of professional services. Our study does not include wave 3 since this wave has a completely different design and purpose than the other waves. In fact, wave 3 provides a retrospective view on participants’ whole life and uses a different set of questions [15]. For each of the waves 1, 2, 4, and 5, we construct measures that are the closest and most comparable with wave 6. In fact, the SHARE structure has developed along the years including more countries and pathologies as well as asking more precise questions on the requirements of formal LTC. For example, the countries Czech Republic, Estonia, Greece and Slovenia included in wave 6 are absent in previous waves. Further, information on mental diseases as well as on rheumatoid arthritis, osteoarthritis, chronic kidney disease and other fractures are not reported in earlier waves. Finally, we notice small differences several questions related to receiving help from professional caregivers. For example, in waves 5 and 6 the question on receiving professional help with domestic tasks specifies “in your own house”, while such specification does not appear in waves 1 and 2. No information on these questions is reported in wave 4. We will not be able to test Conjectures 4 and 5 on historical data since the various regressions include too many different definitions among waves making any results hardly comparable. In Table 9, we report the number *N* of available observations in each wave. Note that the sample size differs throughout the waves, impacting among other the power of our statistical analysis. The detailed results when applying models (1) and (3) on the data of the waves 1, 2, 4 and 5 respectively 1, 2 and 5 are reported in Tables 11 and 12 of the Appendix.

When considering the probability to report ADL limitations, we can validate Conjecture 1a in previous waves. However, it must be noted that the gender coefficient is not significant when the age-gender interaction is included. Such result is unexpected since most research finds significantly higher limitations for men than women at high ages (see our discussion in Sect. 2.2 and the findings in Sect. 4.2). Nevertheless, the age-gender interaction is significant and, when removing that interaction from the model, the gender factor appears to be positive and highly significant as well. While we validate Conjecture 2a among all waves, the LTC scheme has no particular effect on the probability to report ADL limitations yielding rejection of the Conjecture 3a. When considering the probability to report formal LTC usage, we merely find a non-significant effect of the gender leading to partial validation of Conjecture 1b. No conclusion can be drawn on data from wave 4 since formal care has not been recorded that year. Further, we disprove Conjecture 2b in wave 1 (no pathology has a statistically significant effect) and validate it for waves 2 and 5. Note at this point that wave 1 presents less than 1 000 observations on dependent elderly. Finally, for all historical waves, we reject Conjecture 3b since we do not find any effect of the LTC scheme on the probability to report formal care.

The above conclusions are summarized in Table 9. For comparison and as confidence indicator the underlying number *N* of records is indicated for each wave only a lower number of observations is available in earlier waves. Not all factors have the same significance over the 14-years period between waves 1 and 6, but we find similar results along our conjectures with some difficulties to obtain significant results in earlier waves. Although, when challenging our results among waves is subject to bias due to differences in the recorded variables, the analysis performed in this section underlines several findings but also highlights the need of cautiousness in deriving strict conclusions.

**Implications for policymakers and insurers** Our research has significant implications for policymakers and insurers. The number of limitations in ADL is the most standard metric for evaluating the degree of dependence, for checking the eligibility for insurance benefits, and for defining the amount of allowances. Therefore, the description of the factors affecting this measure together with the demographic changes is key for planning government expenses. Among others, our findings show that demographic and medical factors as well as the household composition significantly shape the reporting of ADL limitations. While the whole set of the studied medical factors significantly affect this probability only mental and Parkinson diseases increase the probability for using paid-for services. Such result directly affects the design of LTC insurance (LTCI) solutions that cover observable costs. Further, by knowing that the presence of the partner in the household significantly reduces the usage of formal LTC essentially for domestic tasks, relying only on informal care is not reasonable. Indeed, LTC costs related to personal care delivered by professional caregivers is incompressible and represents an expensive part of LTC. Finally, by observing a significant effect of the specific LTC scheme on the usage of services, our work gives important insights on public-private interactions for LTC financing and for policymaking. In fact, based on our findings, less LTCI crowding out effects are to be expected in countries with family care and subsidiary LTC schemes when compared to countries with State responsibility LTC schemes.

**Limitations of the present study** Our study is subject to a set of limitations. First, the SHARE dataset solely accounts for elderly cared for at home and neglects persons cared for in an institution. Accounting for the latter would bring stronger evidence on the impact of medical factors on ADL limitations since they often report mental diseases. Second, important limitations arise when using a survey setup. As mentioned in Sect. 3.1, the survey is broken into two parts. While general questions are answered under supervision of a purposed-trained employee, more intimate questions and in particular those dealing with pathologies are completed by the respondent while and might thus be more subjective. Third, the SHARE survey has evolved over the years. Thereby, each advancement has brought changes in an important number of questions making cross-wave comparison challenging (see above). Finally, part of the observed relation between ADL limitations and some cofactors might also be subject to endogeneity concerns and collinearity. Given the number of covariates used in our study, collinearity may be responsible for non-significant outcomes. Regarding endogeneity, for example, the value of BMI, mental disorders and wealth can be a consequence as well as a cause of ADL limitations. On the one hand, if a longer observation period is available, the nature of the relationship could be partially clarified by identifying which of the characteristics appear first [22]. On the other hand, additional research could use instrumental variables to eliminate some of the endogeneity arising from the simultaneous determination of ADL limitations and mental diseases [93].

## Conclusion

In this paper, we assess the probability to report ADL limitations and the probability to use formal LTC among elderly aged $65+$ years in Europe. Our study is based on 26 331 individuals from which 3931 report functional limitations and 1470 use professional care services. The data comes from the sixth wave of the SHARE dataset encompassing 13 European countries. We address five conjectures hypothesizing on the importance of demographic, social, medical and policy factors as well as of the ADL and types of formal care in the determination of the aforementioned probabilities.

From a probit regression model, we find that the age, the gender, the BMI, the presence of the partner in the household, the wealth status, the education level and the pathology highly influence the reporting of ADL limitations. First, women aged above 80 years report more limitations than men. Second, while we observe no effect of having children on the reported functional limitations, this result is reversed when a partner lives in the household. Third, persons with higher wealth and higher education level report lower difficulties with ADL. Thereafter, our findings show that pathologies importantly increase functional limitations. However, the effect is lower for people diagnosed with cancer when compared to mental diseases or Parkinson. Finally, the LTC policy of the country of residence has little influence on the reporting.

Based on a logit regression model, we study the covariates influencing the probability to report formal LTC usage. In comparison to functional limitations that can be observed, the usage of professional care is subject to the availability of informal care. In our results, we find that older women appear to require more formal care than men. While some socio-related factors such as the BMI, the smoking habits and the wealth status have no influence, we observe a lower probability for men when there is a partner in the household. This observation highlights the importance of the spouse in providing informal LTC and limiting the demand of professional care. From the pathologies, we find that mental diseases and Parkinson have the highest effect on using formal LTC. In comparison to other diseases, they often require qualified caregivers. Our results also highlight that a cancer diagnosis does not entail significantly more formal care. Further, we observe that elderly living in family care schemes report significantly less formal care usage than those from other cultural backgrounds or countries with different policy arrangements. Finally, by distinguishing formal care along personal and domestic tasks, we find that the partner in the household mostly helps in reducing the probability to demand professional help with domestic tasks.

The SHARE dataset has enabled us to identify functional limitations in ADL and formal LTC usage in a comprehensive cross-country study. While offering an extensive analysis along demographic, social, medical and policy factors, various streams of research could extend the present study. First, the delivery of informal care by relatives surely needs more investigation. For example, providing informal care can also be linked to moral hazard issues in particular linked to the expected inheritance from children (see also [31]). Second, in our study we classified pathologies in five main groups and consider only the main pathology of the elderly, i.e. we do not consider co-morbidity. Knowing that over 12 000 diagnoses are listed in the ICD classification, we are convinced that, with the appropriate data, a more detailed analysis on the importance of pathologies in LTC could be performed (see also [85]). Third, even if we find that including individual SHARE cross-sectional weights does not appear to improve the model, comparing the unweighted approach with models including weights in future waves is recommended since it might help to detect sampling bias (see also [88]). Finally, our findings can foster the development of LTC insurance by giving insights for the underwriting standards used in future products and in different LTC policy environments across Europe. Following this line of reasoning, insights on public-private interactions as well as consequences for designing public LTC schemes can be derived.

## Appendix

See Tables 10, 11, 12, 13, 14 and 15.

**Table 10:** Results for regression model (1) on the probability to report limitations in ADL when including the individual cross-sectional SHARE weights

	Dependent
**Intercept**	$-3.804$ (.327)***
**Age**	0.033 (.004)***
**Gender (baseline: Male)**	
Female	$-1.669$ (.396)***
**Gender** $\times$ **Age**	0.021 (.005)***
**Body mass index (baseline: Normal weight)**	
Underweight	0.574 (.128)***
Overweight	$-0.011$ (.043)
Moderately obese	0.230 (.055)***
Severely obese	0.548 (.086)***
Very severely obese	1.078 (.151)***
**Daily smoker (baseline: No)**	
Yes	0.059 (.040)
**Partner in household (baseline: No)**	
Yes	$-0.142$ (.041)***
**Children (baseline: No)**	
Yes	$-0.040$ (.061)
**Wealth status (baseline: Low)**	
Mid-low	$-0.068$ (.059)
Mid-high	$-0.242$ (.062)***
High	$-0.431$ (.064)***
**Education level (baseline: Primary)**	
Secondary	$-0.040$ (.040)
Tertiary	$-0.263$ (.062)***
**Mental diseases (baseline: No)**	
Yes	0.628 (.056)***
**Parkinson disease (baseline: No)**	
Yes	0.905 (.122)***
**Cancer (baseline: No)**	
Yes	0.293 (.082)***
**Musculoskeletal system diseases (baseline: No)**	
Yes	0.447 (.038)***
**Other physical diseases (baseline: No)**	
Yes	0.357 (.037)***
**LTC scheme (baseline: None)**	
State responsibility	0.070 (.063)
Family care	$-0.017$ (.065)
Subsidiary	0.194 (.063)**
*N*	$26\,331$

$$p < 0.1$$ , *$$p < 0.05$$, **$$p < 0.01$$, ***$$p < 0.001$$

**Table 11:** Results for regression model (1) applied on data from SHARE waves 1, 2, 4 and 5

	Wave 1	Wave 2	Wave 4	Wave 5
**Intercept**	$-7.182$ (.907)***	$-8.228$ (.742)***	$-8.651$ (.521)***	$-8.437$ (.439)***
**Age**	0.083 (.009)***	0.077 (.008)***	0.086 (.006)***	0.085 (.005)***
**Gender (baseline: Male)**				
Female	$-1.187$ (.852)	$-1.455$ (.807).	$-0.844$ (.604)	$-0.449$ (0.509)
**Gender** $\times$ **Age**	0.019 (.011).	0.023 (.010)*	0.013 (.008)	0.008 (.007)
**Body mass index (baseline: Normal weight)**				
Underweight	0.430 (.254).	0.731 (.210)***	0.434 (.182)*	0.508 (.162)**
Overweight	0.216 (.088)*	0.201 (.086)*	0.100 (.065)	0.073 (.056)
Moderately obese	0.647 (.112)***	0.599 (.108)***	0.612 (.081)***	0.469 (.070)***
Severely obese	1.050 (.203)***	1.121 (.181)***	1.115 (.133)***	0.939 (.112)***
Very severely obese	1.793 (.321)***	1.534 (.339)**	1.405 (.234)***	1.584 (.187)***
**Daily smoker (baseline: No)**				
Yes	0.036 (.088)	0.103 (.083)	0.019 (.062)	0.169 (.052)**
**Partner in household (baseline: No)**				
Yes	$-0.161$ (.089).	$-0.192$ (.083)	$-0.223$ (.063)***	$-0.280$ (.053)***
**Children (baseline: No)**				
Yes	$-0.067$ (.107)	0.155 (.111)	$-0.013$ (.085)	0.041 (.075)
**Wealth status (baseline: Low)**				
Mid-low	$-0.075$ (.128)	$-0.268$ (.120)*	$-0.339$ (.097)***	$-0.511$ (.086)***
Mid-high	$-0.448$ (.131)***	$-0.600$ (.122)***	$-0.695$ (.098)***	$-0.819$ (.086)***
High	$-0.410$ (.144)**	$-0.690$ (.136)***	$-1.060$ (.107)***	$-1.113$ (.089)***
**Education level (baseline: Primary)**				
Secondary	$-0.118$ (.087)	$-0.085$ (.083)	0.054 (.064)	$-0.153$ (.057)**
Tertiary	$-0.287$ (.134)*	$-0.244$ (.128).	$-0.187$ (.093)*	$-0.331$ (.079)***
**Parkinson disease (baseline: No)**				
Yes	2.059 (.316)***	2.364 (.277)***	2.346 (.201)***	1.956 (.156)***
**Cancer (baseline: No)**				
Yes	0.282 (.133)*	0.283 (.149).	0.196 (.105).	0.415 (.084)***
**Musculoskeletal system diseases (baseline: No)**				
Yes	1.329 (.163)***	1.104 (.161)***	1.253 (.113)***	1.092 (.104)***
**Other physical diseases (baseline: No)**				
Yes	0.479 (.076)***	0.824 (.074)***	0.715 (.056)***	0.732 (.048)***
**LTC scheme (baseline: None)**				
State responsibility	$-1.247$ (.557)*	$-0.134$ (.293)	0.338 (.118)**	0.243 (.118)*
Family care	$-1.081$ (.560).	0.113 (.297)	0.317 (.126)*	0.352 (.122)**
Subsidiary	$-0.896$ (.555)	0.291 (.289)	0.571 (.114)***	0.599 (.115)***
*N*	$6\,269$	$7\,274$	$11\,975$	$17\,031$

$$p < 0.1$$ , *$$p < 0.05$$, **$$p < 0.01$$, ***$$p < 0.001$$

**Table 12:** Results for regression model (3) applied on data from SHARE waves 1, 2 and 5

	Wave 1	Wave 2	Wave 5
**Intercept**	$-7.922$ (1.730)***	$-7.591$ (1.286)***	$-6.619$ (.746)***
**Age**	0.090 (.013)***	0.088 (.013)***	0.084 (.008)***
**Gender (baseline: Male)**			
Female	0.278 (.421)	0.889 (.494).	0.136 (.281)
**Body mass index (baseline: Normal weight)**			
Underweight	1.524 (.570)**	0.474 (.373)	1.201 (.345)***
Overweight	$-0.168$ (.181)	0.038 (.171)	$-0.160$ (.108)
Moderately obese	$-0.038$ (.223)	0.132 (.212)	$-0.019$ (.132)
Severely obese	$-0.259$ (.401)	0.365 (.322)	0.052 (.199)
Very severely obese	0.518 (.510)	0.185 (.646)	0.405 (.300)
**Daily smoker (baseline: No)**			
Yes	0.075 (.189)	0.011 (.169)	$-0.069$ (.103)
**Partner in household (baseline: No)**			
Yes	$-0.393$ (1.968)	$-1.387$ (1.731)	$-2.993$ (1.015)**
**Partner in household** $\times$ **Age**	$-0.011$ (.025)	0.007 (.022)	0.029 (.013)*
**Partner in household** $\times$ **Gender**	0.339 (.373)	0.122 (.328)	0.623 (.203)**
**Children (baseline: No)**			
Yes	0.205 (.382)	0.233 (.463)	$-0.299$ (.239)
**Children** $\times$ **Gender**	$-0.712$ (.460)	$-0.668$ (.524)	$-0.125$ (.297)
**Wealth status (baseline: Low)**			
Mid-low	$-0.414$ (.258)	0.346 (.229)	0.057 (.156)
Mid-high	$-0.428$ (.264)	0.034 (.233)	$-0.112$ (.158)
High	$-0.519$ (.289).	0.048 (.262)	$-0.100$ (.165)
**Education level (baseline: Primary)**			
Secondary	$-0.505$ (.178)**	0.196 (.165)	0.263 (.110)*
Tertiary	$-0.454$ (.274).	0.614 (.260)*	0.130 (.155)
**Parkinson disease (baseline: No)**			
Yes	0.772 (.451).	1.031 (.372)**	0.543 (.216)*
**Cancer (baseline: No)**			
Yes	$-0.061$ (.259)	$-0.224$ (.276)	$-0.267$ (.156).
**Musculoskeletal system diseases (baseline: No)**			
Yes	0.371 (.269)	0.634 (.251)*	0.329 (.096)***
**Other physical diseases (baseline: No)**			
Yes	0.404 (.159)*	0.334 (.152)*	0.415 (.168)*
**LTC scheme (baseline: None)**			
State responsibility	1.429 (1.256)	$-0.204$ (.564)	0.481 (.242)*
Family care	0.165 (1.265)	$-1.255$ (.571)*	$-0.757$ (.251)**
Subsidiary	1.804 (1.252)	$-0.120$ (.556)	0.611 (.235)**
*N*	983	1092	2542

$$p < 0.1$$, *$$p < 0.05$$, **$$p < 0.01$$, ***$$p < 0.001$$

**Table 13:** Results from the backward step-wise selection process along the Bayesian and the Akaike information criteria (BIC and AIC) for the interactions in models (1) and (3)

Model (1)	BIC	AIC
$AG \cdot GE + AG \cdot HH + AG \cdot CD + GE \cdot SM + GE \cdot HH + GE \cdot CD + HH \cdot CH$	18,623	18,369
$AG \cdot GE + AG \cdot HH + AG \cdot CD + GE \cdot SM + GE \cdot HH + HH \cdot CH$	18,613	18,367
$AG \cdot GE + AG \cdot HH + AG \cdot CD + GE \cdot HH + HH \cdot CH$	18,603	18,366
$AG \cdot GE + AG \cdot HH + GE \cdot HH + HH \cdot CH$	18,593	18,364
$AG \cdot GE + AG \cdot HH + GE \cdot HH$	18,583	18,363
$AG \cdot GE + AG \cdot HH$	18,574	18,362
$AG \cdot GE$	**18,568**	18,364
No interactions	18,596	18,400
Model (3)		
$AG \cdot GE + AG \cdot HH + AG \cdot CD + GE \cdot SM + GE \cdot HH + GE \cdot CD + HH \cdot CH$	4615	4420
$AG \cdot GE + AG \cdot HH + AG \cdot CD + GE \cdot SM + GE \cdot HH + GE \cdot CD$	4609	4420
$AG \cdot HH + AG \cdot CD + GE \cdot SM + GE \cdot HH + GE \cdot CD$	4603	4421
$AG \cdot CD + GE \cdot SM + GE \cdot HH + GE \cdot CD$	4597	4421
$AG \cdot CD + GE \cdot HH + GE \cdot CD$	4592	4422
$GE \cdot HH + GE \cdot CD$	**4590**	4427
No interactions	4597	4446

**Table 14:** Contribution of the variables in models (1) and (3) in percentage of deviance decrease

Variables	Model (1)	Model (3)
Age	6.20	5.73
Gender	0.35	0.97
Body mass index	1.90	0.98
Daily smoker	0.16	0.06
Partner in household	1.55	3.72
Children	0.01	0.98
Wealth status	1.52	0.86
Education level	1.26	0.18
Mental diseases	3.55	0.54
Parkinson disease	1.29	0.20
Cancer	0.19	0.02
Musculoskeletal system diseases	3.87	0.29
Other physical diseases	3.97	0.09
LTC scheme	0.69	5.00
Gender $\times$ Age	0.84	
Partner in household $\times$ Age		3.10
Partner in household $\times$ Gender		0.38
Children $\times$ Gender		0.12

**Table 15:** Confusion matrices for models (1) to (4)

		Model (1)	Model (2)						Model (3)	Model (4)	
Obs.	Pred.	Dependent	Dressing	Walking	Bathing	Eating	In/out of bed	Toileting	Formal LTC	Personal care	Domestic tasks
0	0	16,012	16,754	18,487	18,126	19,137	18,243	18,949	1697	2101	1982
0	1	1122	783	207	538	166	338	214	457	264	331
1	0	6388	6891	7098	6060	6544	6850	6549	764	1027	810
1	1	2809	1903	539	1607	484	900	619	1013	539	808

The columns “Obs.” and “Pred.” stand for observed and predicted values, respectively. The observation-prediction combinations 0–0 and 1–1 correspond to true negatives respectively true positives. Combinations 0–1 and 1–0 account for false positives respectively false negatives. The reported values are obtained by choosing the optimal threshold along sensitivity and specificity model characteristics [58]

## Footnotes

1 The survey methodology highly compares with the one from the Health and Retirement Study (HRS) and the English Longitudinal Study of Ageing (ELSA) reporting information on elderly in the U.S. and England, respectively [74]. The method is assessed to provide reliable and comparable records across European countries [17]. This paper uses data from SHARE waves 1, 2, 3 (SHARELIFE), 4, 5 and 6 [10–15], see [16] for methodological details. For our study, we load the various modules in R programming language and merge them in a single dataframe for each wave.

2 For both probabilities related to ADL limitations and formal LTC usage, we have also tested a linear regression model although such model is less suited for probabilities. In both cases, we find lower values for the in-sample AUC (0.7869 respectively 0.7590) and for the 5-fold cross-validation AUC (0.7854 respectively 0.7614).

3 In the Appendix, see Table 10, we provide the results for model (1) when including individual cross-sectional weights, i.e. the variable denoted *cciw_w6* in SHARE. The results compare to those reported in the first column in Table 7. While we obviously observe changes in the coefficients’ values due to the addition of weights, the significance levels have for most cases not been affected.
